# Extracorporeal Membrane Oxygenation (ECMO) for Acute Respiratory Distress Syndrome (ARDS) During Pregnancy: A Systematic Review

**DOI:** 10.7759/cureus.100730

**Published:** 2026-01-04

**Authors:** Islam Kourampi, Eleni Xourgia, Aristomenis Exadaktylos, Wolf Hautz, Simeon Troesch, Mairi Ziaka

**Affiliations:** 1 Department of Emergency Medicine, University Hospital of Bern (Inselspital), Bern, CHE; 2 Department of Internal Medicine, Bürgerspital Solothurn, Solothurn, CHE; 3 Department of Emergency Medicine, University of Cyprus Medical School, Nicosia, CYP; 4 Department of Medicine, Medic Center Heimberg, Heimberg, CHE

**Keywords:** ards, ecmo, fetal, maternal, pregnancy, survival

## Abstract

Acute respiratory distress syndrome (ARDS) in pregnancy is rare but life-threatening. Extracorporeal membrane oxygenation (ECMO) has been used as rescue therapy in refractory cases, though evidence remains limited. This systematic review aimed to synthesize maternal and neonatal outcomes, describe complications, and evaluate study quality to inform clinical practice and research.

A comprehensive search of MEDLINE, Embase, and Cochrane databases, through May 28, 2025, identified observational studies and case series reporting ECMO use for ARDS during pregnancy or the postpartum period. Two reviewers independently screened studies, extracted data, and assessed risk of bias using the Newcastle-Ottawa Scale (NOS) for cohorts and the Joanna Briggs Institute (JBI) checklist for case series.

Thirteen studies published between 2011 and 2024 were included, most involving venovenous (VV) ECMO. Maternal survival ranged from 33% to 100%, with larger and more recent series reporting survival exceeding 80%. Major complications included bleeding, thromboembolism, infection, and acute kidney injury. Fetal outcomes were strongly influenced by gestational age at ECMO initiation, and neonatal morbidity largely reflected the degree of prematurity. The overall risk of bias was moderate to high across most studies.

In conclusion, ECMO can be lifesaving for pregnant and postpartum patients with refractory ARDS, but current evidence is constrained by observational designs, small sample sizes, and heterogeneity in reporting. Standardized data collection, prospective registries, and collaborative multicenter studies are needed to better define the safety, timing, and long-term outcomes of ECMO use in this unique patient population.

## Introduction and background

Acute respiratory distress syndrome (ARDS) is a critical condition characterized by diffuse alveolar damage, severe hypoxemia, and reduced lung compliance, typically requiring mechanical ventilation, with a mortality rate as high as 40% [1]. Its incidence during pregnancy, though rare, poses unique diagnostic and therapeutic challenges due to physiological alterations in the maternal respiratory and cardiovascular systems, as well as concerns regarding fetal viability. Pregnant women may develop ARDS secondary to infections (influenza/coronavirus disease 2019 (COVID-19)), aspiration, preeclampsia-related complications, or obstetric hemorrhage [2]. Extracorporeal membrane oxygenation (ECMO), particularly venovenous (VV) ECMO, has emerged as a rescue therapy in cases of refractory hypoxemia unresponsive to conventional ventilation strategies [3]. ECMO bypasses the lungs (and, in venoarterial (VA) mode, additionally the heart) to oxygenate blood externally, allowing time for pulmonary recovery [4]. Its application in pregnancy, however, remains controversial and relatively underreported, as maternal hemodynamics, anticoagulation protocols, and fetal safety must all be carefully balanced [5]. Existing literature on ECMO use for ARDS during pregnancy is predominantly composed of case reports, case series, and a limited number of observational studies [5,6]. Although a few systematic reviews have been published, they are either outdated or primarily based on secondary sources rather than original research articles [7,8].

These reviews are often limited by methodological issues, such as small study populations, inconsistent outcome reporting, and limited geographic or temporal representation. Given the increasing prevalence of ARDS in pregnancy and the complex decision-making involved in ECMO use, this systematic review aims to synthesize the latest evidence to provide clinicians with clearer guidance on treatment strategies. 

Physiological changes of pregnancy

**Figure 1:**
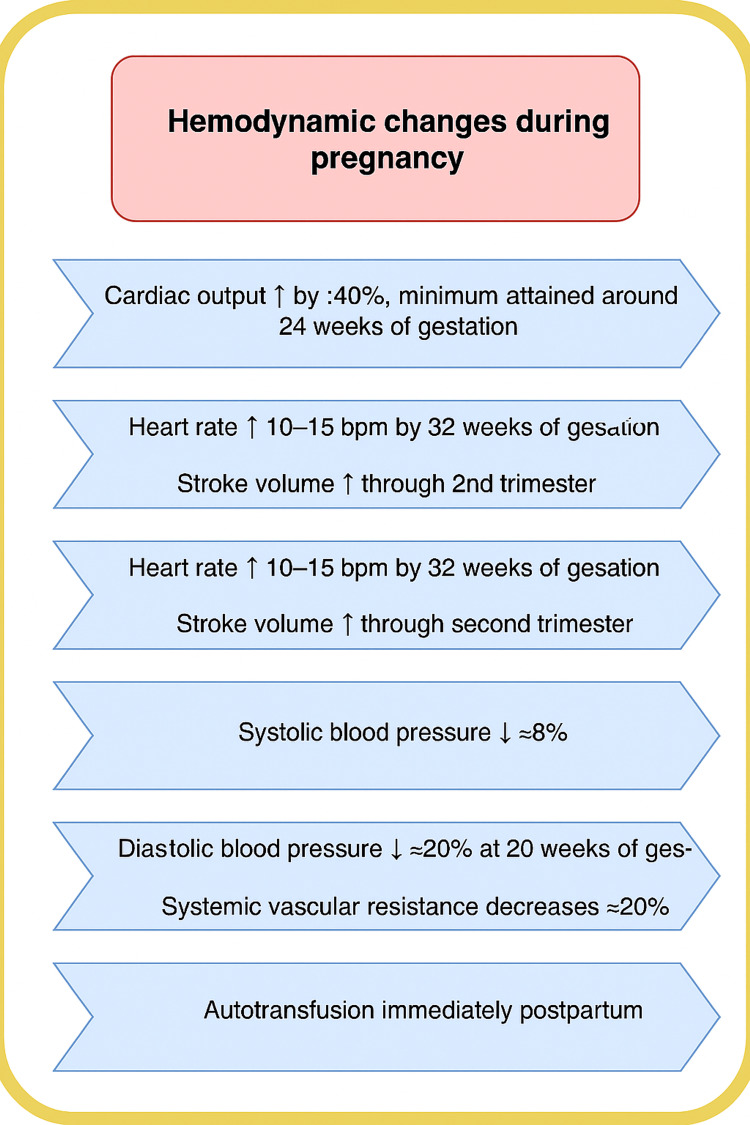
Cardiovascular Changes in Pregnancy

Pregnancy causes major, progressive cardiopulmonary and hemodynamic adaptations that affect tolerance of respiratory failure and the technical management of ECMO. Cardiac output increases by ~30%-40%, peaking near mid-pregnancy (≈24 weeks), largely due to increases in stroke volume and a resting heart-rate rise of about 10-15 beats/min [9]. Systemic vascular resistance falls (≈35%) because of the low-resistance uteroplacental circulation and hormonal vasodilation; in uncomplicated pregnancies, maternal blood pressure typically declines in the second trimester, then returns toward baseline in the third trimester. Despite cardiac enlargement, filling pressures (central venous pressure and pulmonary capillary wedge pressure) generally remain stable because of myocardial adaptation [10]. Hemodynamic changes during pregnancy are summarized in Figure 1.

Changes in the respiratory system are also seen: functional residual capacity is reduced (up to ~20%), minute ventilation increases, and oxygen consumption rises, all of which lower the margin for hypoxemia. These changes increase the risk of rapid desaturation during intubation and complicate ventilatory management in ARDS [11]. In the context of ECMO management during pregnancy, the unique physiological adaptations of gestation carry several practical implications. Hemodynamic shifts, particularly changes in preload and afterload, necessitate individualized decisions regarding cannulation strategy and circuit flow settings, rather than reliance on standard protocols. Anticoagulation also requires special attention, since pregnancy is inherently prothrombotic, yet bleeding risk fluctuates significantly around delivery, making careful adjustment of anticoagulation targets and vigilant monitoring essential. Equally important are fetal considerations, as fetal oxygenation is critically dependent on maternal oxygen delivery and uteroplacental perfusion. This underscores the need for continuous multidisciplinary involvement, structured fetal monitoring, and shared decision-making regarding the optimal timing of delivery. Together, these factors highlight the importance of tailoring ECMO management to the dual needs of mother and fetus, with a coordinated perinatal critical care approach [12]. Because of these complexities, ECMO in pregnancy should be delivered at high-volume centers with multidisciplinary expertise (obstetrics/maternal-fetal medicine (MFM), neonatology, maternal critical care, cardiothoracic surgery/perfusion, anesthesiology, and experienced ECMO nursing and perfusion staff). Early transfer to such centers should be considered when conventional ventilation and adjuncts (prone positioning, inhaled pulmonary vasodilators) fail [13].

## Review

Materials and methods

This systematic review was prospectively registered in the International Prospective Register of Systematic Reviews (PROSPERO; Registration No. CRD420251073254) and conducted in accordance with the Preferred Reporting Items for Systematic Reviews and Meta-Analyses (PRISMA) 2020 guidelines. The review aimed to evaluate maternal and fetal outcomes associated with ECMO in pregnant women diagnosed with ARDS. Eligible participants were pregnant women with ARDS of any etiology who received ECMO support, including both VV and VA configurations. Outcomes of interest included maternal survival and morbidity, fetal survival and neonatal outcomes, intensive care unit (ICU) and hospital length of stay, ECMO-related complications, and gestational age at delivery.

Eligible study designs included case series and observational studies, with no restrictions on publication date, language, or geographic location. Exclusion criteria were applied to case reports, as they often lack the sample size and methodological rigor necessary for drawing generalizable conclusions. Studies that did not provide sufficient data for analysis or that focused on conditions outside the scope of ARDS during pregnancy were also excluded. A comprehensive search of the Medline, Embase, and Cochrane databases was performed, with the final search conducted on May 28, 2025. The search strategy included terms such as “extracorporeal membrane oxygenation,” ECMO, “veno-venous ECMO,” “veno-arterial ECMO,” “cardiopulmonary bypass,” “acute respiratory distress syndrome,” ARDS, “acute lung injury,” “respiratory failure,” “lung failure,” “pregnancy,” “pregnant women,” “gestational,” “maternal,” and “perinatal.” Additional manual searches of reference lists from relevant studies and systematic reviews were conducted to identify grey literature and other potentially eligible articles.

All records were screened independently, in duplicate, by two reviewers (IK and EX). Titles and abstracts were first reviewed for relevance, and full-text articles of potentially eligible studies were then assessed against the inclusion criteria. Any discrepancies were resolved through discussion or consultation with a third reviewer. Data extraction was performed independently by the same reviewers using a standardized and piloted Excel spreadsheet (Microsoft® Corp., Redmond, WA, USA). Risk of bias was assessed according to study design using appropriate critical appraisal tools, specifically the Newcastle-Ottawa Scale (NOS) for observational studies and the Joanna Briggs Institute (JBI) checklists for case series, with detailed assessments presented in Table 1. All assessments were conducted independently, and disagreements were resolved by consensus.

**Table 1:** Study Quality Table

Author and Year	Study Design	Appraisal Tool	Overall Quality Rating
Byrne et al. (2023) [14]	Retrospective cohort study	Newcastle-Ottawa Scale (NOS)	6/9
Malfertheiner et al. (2023) [15]	Retrospective cohort study	Newcastle-Ottawa Scale (NOS)	6/9
Nair et al. (2011) [16]	Retrospective observational study	Newcastle-Ottawa Scale (NOS)	5/9
El Banayosy et al. (2023) [17]	Retrospective, observational study	Newcastle-Ottawa Scale (NOS)	6/9
Aissi et al. (2022) [18]	Retrospective cohort study	Newcastle-Ottawa Scale (NOS) - stars	6/9
Webster et al. (2020) [19]	Case series	JBI Checklist for Case Series	8/10
Sitter et al. (2022) [20]	Prospective cohort study	Newcastle-Ottawa Scale (NOS) - stars	8/9
Kakar et al. (2022) [21]	Case series	JBI Checklist for Case Series	9/10
Piwowarczyk et al. (2023) [22]	Case series	JBI Checklist for Case Series	7/10
Barrantes et al. (2021) [23]	Cross-sectional observational study	JBI Checklist for Case Series - yes	7/9
Clemenza et al. (2022) [24]	Case series	JBI Checklist for Case Series	9/10
Shih et al. (2022) [25]	Case series	JBI Checklist for Case Series	7/10
Bamasood et al. (2022) [26]	Case series	JBI Checklist for Case Series	9/10

Results

**Figure 2:**
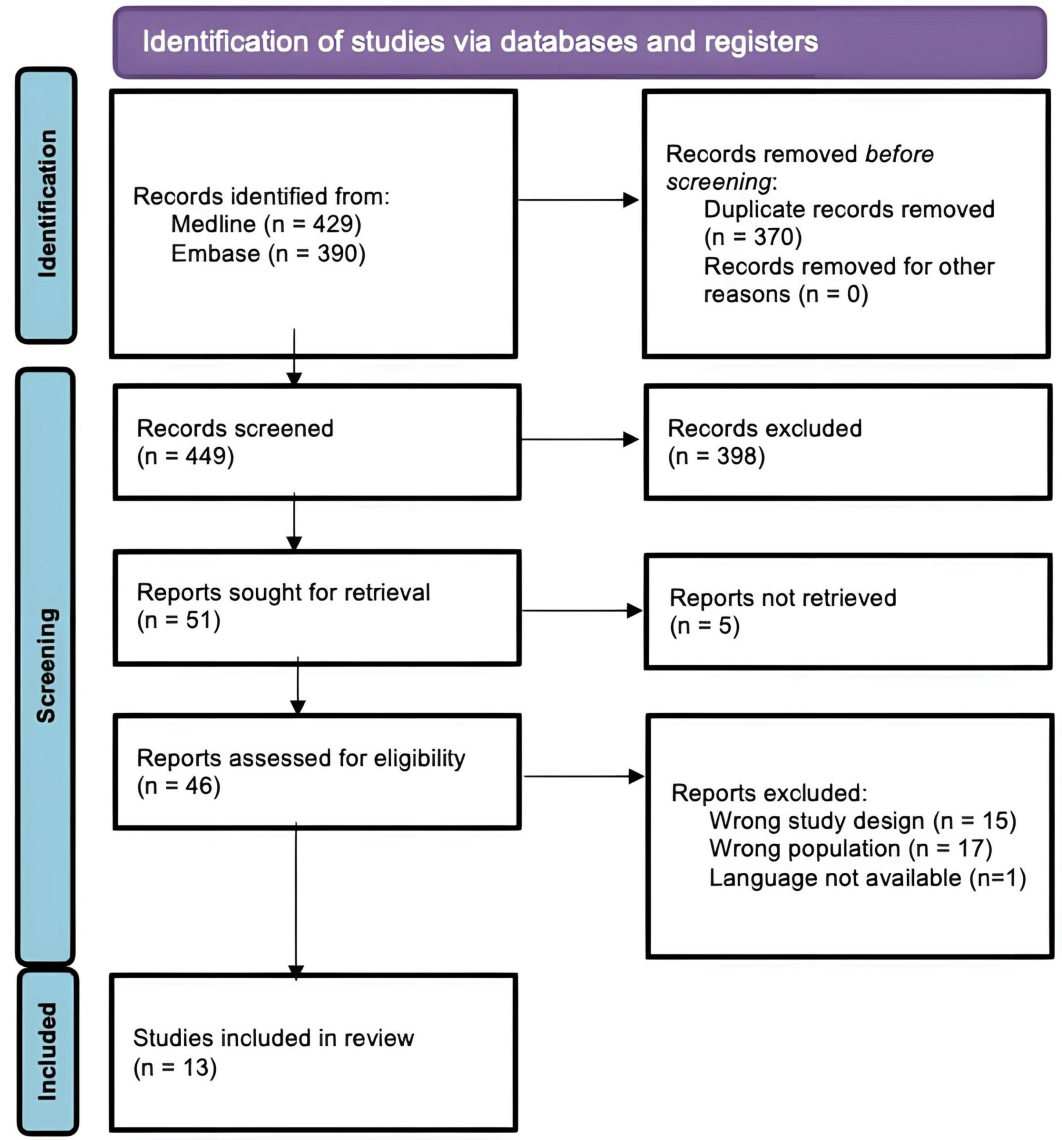
Prisma Flowchart for Systematic Reviews

A total of 819 records were identified through our preliminary search. After removal of duplicates and screening, 13 studies were included in the final synthesis [14-26]. The PRISMA flow diagram is presented in Figure 2.

The included studies (n = 13) consisted of five case series, seven retrospective cohort/observational studies, and one prospective registry-based study, published between 2011 and 2024. Sample sizes ranged from 3 to 60 pregnant or peripartum women with ARDS requiring ECMO. The majority of studies were conducted in high-income settings, including the USA, Europe, and Australia.

Maternal Outcomes

A total of 13 studies (sample sizes ranging from 3 to 60 patients) reported maternal outcomes of ECMO for ARDS during pregnancy or the postpartum period. Mean maternal age across studies ranged between 24 and 43 years. VV ECMO was the predominant mode used, while VA ECMO was applied in a minority of cases. The proportion of patients receiving ECMO during pregnancy versus postpartum varied substantially: some studies included exclusively postpartum patients, while others reported a mix, with initiation typically in the third trimester or postpartum period. The duration of ECMO support ranged from a median of four days to a maximum of 143 days. Maternal survival rates varied widely, from 33% in a small U.S. case series [19] to 100% in several single-center cohorts [17,21,23]. Commonly reported complications included bleeding (up to 67%), thromboembolic events, acute kidney injury, cardiac complications, and infections. In some studies, morbidity exceeded 70%, even in survivors. Higher obstetrical major bleeding was reported after postpartum ECMO (46% vs 18%, p = 0.05) [18]. A few small series reported no major complications. Maternal outcomes are summarized in Table 2.

**Table 2:** Maternal Outcomes of ECMO for ARDS During Pregnancy/Postpartum VV ECMO, Veno-venous extracorporeal membrane oxygenation; VA ECMO, Veno-arterial extracorporeal membrane oxygenation; VVA ECMO, Veno-venoarterial extracorporeal membrane oxygenation; ICU, Intensive care unit; AKI, Acute kidney injury; VTE, Venous thromboembolism; VAC therapy, Vacuum-assisted closure therapy; COVID-19, Coronavirus disease 2019; ARDS, Acute respiratory distress syndrome

Author and Year	Country	Study Design	Sample Size	Mean Age	ECMO Type	ECMO Timing	Duration (Days)	Maternal Survival	Major Complications
Byrne et al. (2023) [14]	USA	Retrospective cohort study	100 (47 during pregnancy)	31.1 years	VV and VA ECMO	25.1 weeks	Median 20	84%	76% had morbidity (VTE, AKI, cardiac)
Malfertheiner et al. (2023) [15]	Multinational	Retrospective cohort study	30	30.5 years	77% VV, 23% VA	Majority postpartum	Not stated	87%	Bleeding, thrombotic events
Nair et al. (2011) [16]	Australia and New Zealand	Retrospective observational study	12	29 years	10 VV, 2 VA	All 2nd/3rd trimester	Median 14	66%	Bleeding (67%), infections
El Banayosy et al. (2023) [17]	USA	Retrospective observational study	8	31 ± 4 years	7 VV, 1 VA	2 pregnant, 6 postpartum	7-74 ICU days	100%	Bleeding (63%), 1 hysterectomy
Aissi et al. (2022) [18]	France	Retrospective cohort study	24	33 years	VV (except 1 VA)	Mixed	20-33	73-85%	More bleeding if postpartum
Webster et al. (2020) [19]	USA	Case series	9	24 years	8 VV, 1 VA	4 pregnant, 4 postpartum	Median 6	33%	None reported
Sitter et al. (2022) [20]	Germany	Prospective cohort study	15	33 years	14 VV, 1 VA	Mostly 3rd trimester	Median 25	87%	Bleeding, thromboembolism
Kakar et al. (2022) [21]	UAE	Case Series	5	-	VV	1 pregnant, 4 postpartum	4-19	100%	None reported
Piwowarczyk et al. (2023) [22]	Poland	Retrospective case series	5	27-39	VV	All postpartum	Median 11	40%	No thrombotic events
Barrantes et al. (2020) [23]	USA	Cross-sectional study	9	30 years	VV	2 pregnant, 2 peripartum	Median 10	100%	Mild bleeding, no major issues
Clemenza et al. (2022) [24]	Italy	Case series	3	27-43 years	VV	2 pregnant, 1 peripartum	20-143	2 survived	Sepsis, VAC therapy
Shih et al. (2022) [25]	USA	Case series	10	30 years	VV	2nd trimester, postpartum	Median 22	60%	Hemorrhagic stroke, ischemic stroke, bleeding
Bamasood et al. (2022) [26]	Kuwait	Case series	10	32 years	8 VV, 2 VVA	3rd trimester	2-20	90%	1 septic shock

Fetal and Neonatal Outcomes

Fetal survival rates ranged from 55% to 100%, with the lowest survival associated with ECMO initiation during pregnancy before 30 weeks’ gestation. Preterm delivery was frequent, with median gestational ages at delivery often below 32 weeks, and some reports noted delivery before 28 weeks. Delivery mode was predominantly cesarean section when reported, though a few vaginal deliveries occurred [18,19,24]. Neonatal intensive care unit (NICU) admission rates were high (up to 86%), largely reflecting prematurity. Reported neonatal complications included bronchopulmonary dysplasia, respiratory distress syndrome, low Apgar scores, hydrocephalus, and isolated cases of sepsis. Fetal demise was often associated with extreme prematurity or maternal death. No congenital anomalies were consistently reported, and vertical transmission of infections (e.g., COVID-19) was rare [24]. Overall, maternal survival was generally favorable in larger [14,15], more recent series, while fetal outcomes were heavily influenced by gestational age at ECMO initiation, with the poorest outcomes seen in pregnancies <30 weeks at the time of support [16,19,25]. Fetal and neonatal outcomes are summarized in Table 3.

**Table 3:** Fetal/Neonatal Outcomes of ECMO for ARDS During Pregnancy/Postpartum NICU, Neonatal intensive care unit; RDS, Respiratory distress syndrome; CS, Cesarean section; IUFD, Intrauterine fetal demise; ECMO, Extracorporeal membrane oxygenation; ARDS, Acute respiratory distress syndrome

Author (Year)	Fetal Survival	Gestational Age at Delivery	Delivery Mode	NICU Admission	Complications	Notes
Byrne et al. (2023) [14]	55.3%	Median 29.4 weeks	Not stated	86.2%	Respiratory distress syndrome	80% low birth weight
Malfertheiner et al. (2023) [15]	73%	<30 weeks mostly	Not stated	High	Feta acidosis, hypoxia	Fetal deaths >30 weeks → no association with ECMO
Nair et al. (2011) [16]	71%	Median 31 weeks	CS	80%	Low Apgar, 2 stillbirths	No anomalies
El Banayosy et al. (2023) [17]	100%	Not stated	100% CS	None	None	No complications reported
Aissi et al. (2022) [18]	55-92%	<28 weeks in some	CS/vaginal	Moderate	2 neonatal deaths	Higher survival post-delivery ECMO
Webster et al. (2020) [19]	60%	Varies	Mixed	Not stated	Previable losses	All losses with maternal death
Sitter et al. (2022) [20]	91%	Mostly preterm	Not stated	49 neonates in NICU	RDS	No neonatal deaths
Kakar et al. (2022) [21]	2 losses	Not fully stated	Not stated	Unknown	1 fetal demise before ECMO	Neonatal info incomplete
Piwowarczyk et al.(2023) [22]	80%	3rd trimester	CS	Some	Hydrocephalus, RDS	All COVID-negative
Barrantes et al. (2020) [23]	89%	23-37 weeks	CS	Most	1 neonatal death	No vertical transmission
Clemenza et al. (2022) [24]	100% (2/3)	31-38 weeks	2 CS, 1 vaginal	2 NICU	1 tested COVID+	All good outcomes
Shih et al. (2022) [25]	80%	24-28 weeks	CS	-	-	-
Bamasood et al. (2022) [26]	90%	33 weeks	Not stated	1	1 IUFD	-

Discussion

Although the management of critically ill pregnant and postpartum women in the ICU is relatively rare, ranging from 0.7 to 13.5 per 1,000 deliveries [27], mortality remains high, ranging from 6.5% to 12.4% in high- and low-middle-income countries, respectively [28]. The main obstetric indications for ICU admission include hypertensive disorders of pregnancy, hemorrhage, HELLP syndrome (hemolysis, elevated liver enzymes, and low platelet count), and acute fatty liver of pregnancy, whereas non-obstetric indications comprise cardiac disorders such as peripartum cardiomyopathy, sepsis, and malignant tumors [29,30]. Notably, more than half of these patients necessitate mechanical ventilation [31]. ARDS has traditionally been considered a rare complication, occurring in 0.05% to 0.3% of pregnancies. However, recent outbreaks of viral infections, including H1N1 influenza and COVID-19, have made pregnant women an important ARDS subpopulation, particularly given the high morbidity and mortality rates of up to 60% [2,32,33]. Pregnant individuals are at increased risk of severe respiratory compromise due to physiological adaptations such as reduced functional residual capacity and increased oxygen consumption [34]. ECMO has emerged as a critical rescue modality for managing ARDS during pregnancy, particularly when conventional therapies fail, with maternal survival rates in the reviewed studies substantially higher than historic mortality for severe ARDS without ECMO [35].

In our study, the duration of ECMO support ranged from a median of four days to a maximum of 143 days. These findings are consistent with studies investigating ECMO support in non-pregnant patients with ARDS, which have reported typical durations of one to two weeks. However, prolonged ECMO support, defined as lasting more than three weeks, has also been described [36]. Similarly, we observed cases of prolonged ECMO support, including one exceptionally long case of 143 days in an obese pregnant patient with COVID-19 [24]. In the systematic review by Palella et al. on pregnant women with ARDS, the mean duration of ECMO support was 19.1 days, and more than half of the patients underwent prone positioning prior to ECMO initiation [7]. Reported maternal mortality was 16.9%, corresponding to a survival rate of 83.1%. Fetal mortality was 31.8%, with poor neonatal outcomes largely attributed to complications of prematurity [8]. We found wide variations in maternal mortality, with reported survival rates ranging from 33% in a small case series to 100% in several single-center cohorts. Commonly reported complications included bleeding, thromboembolic events, acute kidney injury, cardiac complications, and infections.

A key insight from the study by Aissi et al. is the association between delivery timing and neonatal outcomes [18]. While maternal survival was similar whether ECMO was started during pregnancy or postpartum, fetal survival was significantly improved when delivery preceded ECMO initiation. However, maintaining pregnancy during ECMO reduced risks of extreme prematurity in survivors, suggesting a complex trade-off that requires individualized, multidisciplinary decision-making. In our study, fetal survival rates varied, ranging from 55% to 100%, with the lowest survival associated with ECMO initiation before 30 weeks’ gestation. Notably, preterm delivery, frequently before 32 or even 28 weeks, was common, mainly via cesarean section, although isolated vaginal deliveries were also performed [18,19,24]. In our study, prematurity was identified as a risk factor for NICU admission, with reported complications including bronchopulmonary dysplasia, respiratory distress syndrome, low Apgar scores, hydrocephalus, and isolated cases of sepsis. Our findings are consistent with previous research highlighting prematurity as a risk factor for NICU admission [37,38] and for the development of complications such as hypoglycemia, hypothermia, hyperbilirubinemia, and respiratory morbidity [37]. Notably, the incidence of respiratory distress syndrome is inversely associated with gestational age, with reported rates of 9% at 34 weeks and 0% at 40 weeks of gestation [37]. The reviewed evidence supports the transfer of pregnant and postpartum ECMO candidates to high-volume, experienced centers with the capacity for emergent delivery. Decisions about delivery timing should balance maternal oxygenation needs against the risks of extreme prematurity. ECMO should be considered early when conventional ventilation fails, especially in COVID-19 ARDS, where prone positioning may be less feasible.

Limitations of the evidence

All included studies are retrospective case series or observational cohorts, generally limited by small sample sizes and a likely publication bias toward favorable outcomes. To mitigate these limitations, we synthesized data across centers and time periods, providing a broader overview than single reports alone. Patient selection, thresholds for ECMO initiation, and anticoagulation protocols varied considerably, limiting direct comparability. Long-term neurodevelopmental outcomes in neonates remain under-reported; by highlighting this gap, our review underscores the need for standardized follow-up in future studies. Several included reports focused specifically on COVID-19-associated ARDS, although the underlying trigger is not expected to fundamentally alter ECMO-related physiology [14,17,23,25].

## Conclusions

Current evidence suggests ECMO is feasible and often effective in the management of ARDS during pregnancy, but maternal morbidity remains considerable, and neonatal neurodevelopmental outcomes are insufficiently reported. While survival rates are encouraging, the reliance on retrospective case series, small samples, and non-standardized protocols hampers definitive conclusions. Our review highlights the urgent need for prospective multicenter registries, standardized definitions of maternal and neonatal outcomes, and structured long-term follow-up to establish evidence-based recommendations for this high-risk population.
